# Dr. Ned’s Reflections

**Published:** 1876-01

**Authors:** 


### DR. NED'S REFLECTIONS.

To many, mankind appears to be nothing more than a mysterious sort of machine, set up and put in motion only to make money. Others look upon this crowning glory of creative wisdom with about as much interest as they would a crib to contain products of a corn field, and put on stilts so as to cheat rats and mice out of an honest living. Others again, regard him as a convenient and well made frame, designed to support a stomach in which sausage, lager beer, fighting whiskey, lobster salad and delicious meats can be put away and no charge for storage demanded. Some look upon a human being as an imperfect muscular appendage to a mouth, which, in the absence of more convenient means, is used to spit tobacco within two feet of a spittoon, in every direction, to save carpets from moth. A larger class can see in the race fine specimens of animal creation controlled, to appearances, by instincts common to all brutes, able to walk, work, grow, digest, absorb and die.

A class, perhaps, far more numerous than all the others combined, seem to act upon the theory that stomachs are mills to grind so much each day, and that men are millers whose first duty it is to attend to the hopper (or mouth,) in which they turn grist after grist for disease and death to toll. Sick or well, faint or strong, the mill must go, or, in their judgment, the body will perish, for to them it is evident that the stomach is the organ in which they "live, move and have being."

I have seen those who acted as if they ran their stomachs by water. Be it known that the material is dissolved by a special agent and not ground in this viscus, hence the more fluids taken with the food, the more diluted is the gastric juice, and the slower must be the process.

Stomachs, limbs, absorbents, &c., are all essential to the economy, but should not be regarded as holding supreme sway.

Stomachs are excellent things to have, if properly treated, but very poor companions when out of humor. Limbs are first-class articles and should be regarded with especial favor if free from bunions, provided that they do not carry us into trouble and then refuse to run us out. Absorbents have their value and should be encouraged in every laudable enterprise, but great care should be taken lest they absorb too much, or contract the infamous habit of taking what does not properly belong to them. So we could go through the various tissues and organs, giving to each its merit, admiring each in its operation and precision, until struck with astonishment, we are forced to ask where lies the hidden spring that animates and drives on these varied forces or systems? When the mighty mystery is unraveled, we are forced to the conclusion that the supreme control of the delicate and complex organisms which constitute the human body, is vested in the mind or nervous system, or the brain and its branches, or, we might say, in the vast battery within the skull, with its endless telegraphic lines, neatly adjusted, each one perfectly insulated and composed of different matter, stretching to every organ and atom of the body, having on one side a white material to carry intelligence from the brain, and on the other a gray substance to convey to the main depot the news from the outer world; for example, one of the main lines goes to the organ of vision, which without hesitation obeys the mandate issued at headquarters, and the eye looks out upon the hills, mountains, valleys, lakes, streamlets, rivers, cataracts, clouds, stars, or sun, and sends intelligence of them entire to the brain, to be photographed and stored away within its wondrous labyrinths, until the demand is made, when instantly the grand treasure-house is unlocked and the pages of memory read and rehearsed. Other lines extend to the extremities, the mind wills the limb to act, the will and the execution of the order, as well as the information of obedience to the main battery, is all instantaneous; no halting to be observed here, no waiting for a reply is ever known while the lines are up or in order; but sever the connection, and the lines that but a moment ago were so indispensable, becomes a clog and burden to us.

We might produce similar illustrations to an indefinite extent, showing the action of this system, stretching through all the senses and organs, operating independent of us, to sustain life, and under our commands, to render life beautiful and interesting; but enough has been said to show us that philosophy and science must admit the fact, that mind reigns as supreme in the body as the sun does in the heavens, and there lies, without doubt, before the real student, vast fields as yet untouched and unexplored, as well as an ample chance to demonstrate and reduce to practical utility the influence exerted upon the physical nature for good or evil, by this mysterious, imponderable, unseen agency, called mind.

We frequently hear of marvellous cures being produced in a moment, and that too in cases which have long resisted the most scientific and skillful remedial means. In such cases, the medical world is inclined to sneer, and always swift to denounce as the "extreme of quackery," while the sufferer gets well and relishes the deception. If the recovery arises from some powerful moral cause, it is denominated "only chance." Say what we will, a cure has in some way resulted, and the force which produced it must be somewhere, it may be that it lay dormant until accident summoned it to the contest. All this being true, it becomes the province of the real student to fathom the mystery if he can, and learn this hidden force, instead of making it a subject of ridicule or contempt, and I am satisfied that if we will desist from our controversies about pathies and isms, and become students, manly, fearless investigators, we will soon agree with Dr. Rush, who asserted that there are "laws of the animal economy which have no place in the systems of nosology or the theories of physics."

To ignore everything new is the province of the fool or Pharisee, but to investigate and prove all things, is the first duty of the true philosopher.

All physicians agree that the mind is capable of developing disease, and all know that a sudden and powerful impression does sometimes eradicate the malady. All know that so called curative agents will produce disease in persons who are well. If the principle holds good in one instance, why should it be denounced in all others?

It is not my object to establish or defend any theory, but simply to throw out some hints, hoping to induce in others, a spirit of investigation and research. And yet, I have no doubt that the strange phenomenon attributed to mesmerism, psychology, clairvoyance or spiritualism, will some day be more perfectly understood, and perhaps made swift and powerful agents or adjuncts, in the treatment of a vast variety of diseases.

It is beyond dispute that mental influences do produce, as well as cure, physical disorders. It is a fact in medical history that the ague, which is said to be the result only of miasma, has been induced by mental excitement, and in the "Annales Medico Psychologiques," a case of Tertian fever is reported, as the product of a vivid normal emotion. The subject saw a person throw himself from a house-top, and was seized at once with nervous tremors which became alarming, and only yielded to the most energetic treatment to give place to the ague, with all its peculiar train of symptoms, bidding defiance to the best medical skill, until at last a brother, supposed to have been ship-wrecked, suddenly put in an appearance and the joy was so intense that the "ague never returned."

If I should chance to have an inveterate case of the ague in my own person, I should be exceedingly delighted to change quinine for a lost brother, and throw the disorder into the bargain, if any one would accept the boot money.

The Duke of Norfolk "fell into an ague" when he ascertained that Queen Elizabeth suspected him of conspiracy. At one time in medical history a permanent remedy for intermittent fever was, to wear around the neck in some way, the word "Abracadabra." Pieces of halter or chips from the gallows, were once supposed curatives when so worn. Galen even had great faith in the use of Amulets in ague, and Ferrarius is reported as having cured fifty cases in a few months by giving to each of his patients a slip of paper on which was written the word "febrifugo." They were directed to cut off one letter each day. The case required only nine days of trusting and cutting. John Hunter boldly affirms that "agues have been cured by charms." Now if these things are true, let us push forward with vigor, benefit our fellows, and advise our patients to take charms instead of Peruvian Bark or Arsenic.

Universal anasarca has been known to be produced in one night, as a result of violent mental emotion.

John Ford, of the Royal Navy, (says an English author), was discharged from the Navy Hospital as an incurable and hopeless case of dropsy. A physician of some distinction suggested to him the propriety of a surgical operation as a last resort. It produced so much alarm and excitement that he broke out into a profuse perspiration, which entirely eradicated his disease and allowed him to return to his duty.

Persons have been so alarmed as to sweat blood, a case of which is detailed by Pauline, in the person of a sailor. Many instances are on record in which, from intense excitement, the hair has become white in a few hours, and has been known to assume its former color under opposite circumstances. The "Lancet" of May 4, 1867, mentions the case of a man who lost all his hair, even his eye-lashes and eye-brows, of a result of anxiety concerning pecuniary losses. At length, a profitable enterprise offered, in which he engaged, and with his returning fortune, his hair grew as prolific as ever. After a time he had another reverse; again his hair fell off. I have heard of a country so poor that hens could not lay eggs, but never before heard of a man so reduced in financial affairs that his hair could not grow, or so prosperous in business that he could hide a naked scalp with anything short of "store hair." If business does thus effect the hair-growing enterprise, I must say that the bare-headed citizens of this country have had a poor chance for the past few years. Now, the times are growing brighter, let the bald take new hope, for hair-workers and wig-makers will have nothing to do when business revives, and the currency question becomes fairly settled.

NED.

---

VENGEANCE ON HAMLIN.—A man in Nebraska, who dropped two cents' worth of mail matter in the post-office box, and had to pay six cents to do it, went over and stood by one of the windows and said: "May Hannibal Hamlin, of Maine, have the bilious colic, the ague, the gout, the jaundice, corns, bunions, boils, and the buckwheat scratches, from this day noon, for the next fifty years to come." And every one within hearing cried amen!

---

CURIOUS BLINDNESS.—An Irish oculist, having completely restored a woman's sight, related, with astonishment, that while she could thread the finest needle, she couldn't tell one letter from another. "She had the same difficulty," replied her attending physician, "before her sight was impaired, as she never went to school."

